# Socio-demographic and socio-economic differences in the availability of green space in the Netherlands

**DOI:** 10.1007/s11111-025-00498-3

**Published:** 2025-06-23

**Authors:** Mingwei Liu, Erik J. Timmermans, Alfred Wagtendonk, Paul Meijer, Diederick E. Grobbee, Ilonca Vaartjes

**Affiliations:** 1 Julius Center for Health Sciences and Primary Care, University Medical Center Utrecht, Utrecht University, Utrecht, Str6. 131, P.O. Box 85500, 3508 GA The Netherlands; 2 Department of Epidemiology and Data Science, Amsterdam UMC, Vrije Universiteit Amsterdam, Amsterdam, The Netherlands

**Keywords:** Green space, Health equity, Socioeconomic status, Ethnic disparity

## Abstract

We aimed to map the spatial distribution of green space in the Netherlands, and to first comprehensively assess socio-demographic and socio-economic differences in the availability of green space in the Netherlands. Data was analyzed from all registered residents of the Netherlands aged one and above on January 1, 2017 (16,440,620 individuals). Socio-demographic and socio-economic information was derived from Statistics Netherlands. Data on green space density (percent of the area) around home were assembled by the Institute for Public Health and the Environment at the address level. The distribution of exposure to green space was described by age groups, sex, ethnicity, household socioeconomic status (SES), and urbanicity degree, and stratified by green space type. The distribution of green space by all vegetation, trees, shrubs, low vegetation, grass field, and agriculture was mapped for the Netherlands in 2017. Small differences in green space density were found across age and sex groups. Ethnic Dutch (58.1%) and Indonesian (54.5%) had more green space coverage around residence than Turkish (50.0%) and Moroccan (50.0%). People with higher household SES generally had greater green space coverage, although it slightly decreased at the highest SES level. Higher urbanicity levels were monotonously associated with lower green space exposure. These differences particularly originated from differences in low vegetation. Observed differences between ethnic and SES groups originated mostly from differences in rural to moderately urbanized areas. Environmental injustice was found among ethnic, SES, and urbanicity groups. The differences were mostly present in low vegetation and in rural to moderately urbanized areas.

**Supplementary Information:**

The online version contains supplementary material available at 10.1007/s11111-025-00498-3.

## Introduction

Since the Warren County movement in 1982 in the USA when civilians protested against dumping contaminated soil in the county with the highest proportion of African Americans, environmental racism has become a political and academic hotspot (Mohai et al., 2009). As social movements and research developed, the concern grew beyond ethnic differences in exposure to environmental hazards (Mohai et al., 2009). The paradigm shifted to environmental equality and, more inclusive, environmental justice (Holifield, 2001; Mohai et al., 2009). The environmental justice framework embraces the principle that all people regardless of race, colour, national origin, or income are entitled to equal distribution of environmental amenities and no group should be disproportionately affected by environmental hazards (Mohai et al., 2009). The environmental justice has become a global concern since early 2000 s (Mohai et al., 2009).

Green space exposure has been associated with multiple health benefits, including improved mental health, and lower risk of obesity, cardiometabolic diseases, and mortality (Ccami-Bernal et al., 2023; Liu et al., 2023; Markevych et al., 2017). There are four potential pathways linking green space exposure to health benefits (Markevych et al., 2017). First, green space may encourage an active lifestyle (Markevych et al., 2017), which in turn is beneficial to people’s overall health status (Warburton & Bredin, 2017). Second, green space may reduce physiological stress and improve mental health (Thompson et al., 2012). Third, green space can reduce harm from exposure to air pollution, heat, and noise (Markevych et al., 2017). Last, green space releases certain chemical agents like phytoncides that may inhibit inflammation (Day, 2012).

Different types of green space may function differently in these four pathways (Markevych et al., 2017). For example, a study in New York City found better self-reported health in people with higher tree density, but not in people with higher grass density (Reid et al., 2017). Another study in Doetinchem, a town in the eastern Netherlands, found that more agricultural land was associated with lower levels of physical activity, while more urban green space was associated with higher levels of physical activity (Picavet et al., 2016). Nevertheless, in the Netherlands, parts of agricultural land can indeed be actively used by residents as walking and/or biking routes (“Fietsknoop”, 2024; “Klompenpaden; de mooiste wandelroutes” 2024). Therefore, agriculture was included as a type of green space in the current study.

### Literature review

Some previous studies investigated the environmental justice of green space exposure and yielded inconsistent findings in different settings. Dobb et al. analyzed 100 big cities around the world (Dobbs et al., 2017). They found that there was less green space in cities that were characterized by a higher urbanization grade (Dobbs et al., 2017). Generally, previous studies found that higher socioeconomic status (SES) levels were associated with more green space, including evidence from Europe (Schüle et al., 2019), USA (Klompmaker et al., 2023; Yi Sun et al., 2021; Wen et al., 2013), Canada (Pinault et al., 2021), and China (P. Sun & Lu, 2022; Yu et al., 2020; Zhuang et al., 2023). Only two studies from the USA were nationally representative (Klompmaker et al., 2023; Wen et al., 2013). Previous evidence was mostly derived from ecological studies that were on small area levels like neighbourhood, community, and census tract. On the contrary, Ju and colleagues found that cities or sub-cities with higher SES had less green space in Latin America (Ju et al., 2021). This was possibly explained by the confounding effect of urbanization, which was associated with higher SES and lower green space exposure (Ju et al., 2021). A previous nationwide study in the USA found urban–rural differences in green space exposure by poverty levels (Wen et al., 2013). In this study, poverty levels were negatively associated with green space exposures in urban and suburban areas while these were positively associated in rural areas (Wen et al., 2013).

Compared to socioeconomic differences in the availability of green space, ethnic differences have been less extensively studied (Yan Sun et al., 2022). A US nationwide study in 2006–2010 found that, on average, census tracts with higher percentages of African Americans and Hispanics included less green space (Wen et al., 2013). A US nationwide study in 2015–2019 found that census tracts with higher percentages of non-Hispanic Whites and lower percentages of Hispanics included, on average more green space (Klompmaker et al., 2023). A weak positive association was observed between percentages of non-Hispanic Blacks and green space exposures in urban tracts but not in rural tracts (Klompmaker et al., 2023). A Canadian study that focused on urban environments found that minority ethnic populations (i.e., Latin American, African Canadian, and South Asian particularly individuals of Filipino ancestry) were exposed to less green space than the majority population (i.e., Whites) (Pinault et al., 2021).

However, previous results are not generalizable to Western European countries, including the Netherlands, because of differences in ethnicity and socio-economic composition, built environment, lifestyle, and patterns of residence (Rodrigue, 2020). For example, as of 2021, 25.4% of the population had a migration background (Statistics Netherlands, 2025). The current unique ethnic composition originated from the history that, from the 1960 s to the 1970 s, the booming Dutch economy encouraged guest workers from Morocco and Turkey (Boffi, 2024). And from the 1980 s to the 2000 s, the government welcomed people from former Dutch colonies (Dutch Caribbean and Suriname) and refugees from eastern European countries (Boffi, 2024). These immigrants tended to reside together (Zorlu & Mulder, 2007) and might have different environmental exposure than ethnic Dutch.

Previous studies in the Netherlands predominately focused on green space exposures by income groups (Kruize, 2007; Kruize et al., 2007a, b; van Velzen & Helbich, 2023), but did not comprehensively include socio-demographic factors, other socio-economic factors, and a variety of vegetation types. But policymakers are increasingly interested in the distribution of green space by vegetation types, as different types of vegetation have varying health effects (Markevych et al., 2017). However, currently, such comprehensive national assessment in the Netherlands is lacking.

### Objectives

The present study aims to address this research gap by (i) determining the spatial distribution of green space by mapping it on neighbourhood level in the Netherlands; and (ii) assessing whether green space exposure differs across socio-demographic and socio-economic subgroups at the individual level.

## Method

### Data source and linkage

A cohort of all registered residents of the Netherlands aged one and above on January 1, 2017 (*n* = 17,074,889) was built from the National Population Register (Prins, 2017). The register contains information on all legally residing citizens in the Netherlands, including date of birth, sex, current and previous residential address, and nationality (Prins, 2017). Information on SES was integrated by Statistics Netherlands (CBS) and originated from the National Population Statistics, the Integrated Income and Assets Survey, the Employment and Wages Statistics, and the Education Level File (“SES-WOA scores per district and neighborhood” 2025). A detailed description of the data sources can be found elsewhere (Statistics Netherlands, 2024a). Area-level exposure data on green space and neighbourhood urbanicity were obtained from the Geoscience and Health Cohort Consortium (GECCO) (Lakerveld et al., 2020; Timmermans et al., 2018). These area-level exposure data were linked to individual-level data (age, sex, ethnicity, and household income) of all residents based on their residential address on January 1, 2017 in the secure environment of CBS. Linkage was successful for 96.3% of the addresses, and unsuccessful linkages were due to inconsistencies in addresses. The analytical sample included 16,440,620 residents. All data linkages and analyses were conducted in line with the policy from CBS and privacy legislation in the Netherlands. Ethical approval was not required for the present study.

### Measures

Data on residential green space was assessed as green space density, which refers to the percentage of an area devoted to green space including trees, shrubs and low vegetation (i.e., grass field and agriculture) within a Euclidean buffer with radii of 500, 1000, and 2000 m around residential addresses. These buffer zone sizes are often applied in environmental health studies, and are considered to be relevant spatial contexts for behaviours and health of people (Y. Liu et al., 2025). The data were assembled by the National Institute for Public Health and the Environment (RIVM) (“National Institute for Public Health and the Environment” 2025) at the address level on a 10 m × 10 m resolution in 2017. Low vegetation data from RIVM is based on satellite-derived altitude data. When using these data from RIVM, agriculture density can be underestimated because of seasonal change and fallow period. Therefore, the individual-level agriculture density was separately derived from another source, namely the CBS land use map 2017. For constructing the neighbourhood-level map for agriculture, a third source, the Basic Registration of Crop Parcels depicting the polygons of agriculture, was used (“Dataset: Basic Registration of Crop Parcels (BRP)” 2024).

Based on age in years, individuals in the analytical sample were categorized into children (1 to 11 years), adolescents (12 to 17 years), adults (18 to 64 years), and older adults (≥ 65 years). The largest four ethnicity groups in the Netherlands were included, which are Dutch (77.9%), Turkish (2.3%), Moroccan (2.2%), and Indonesian (2.1%) based on the National Population Register (Prins, 2017). Household SES was assessed by the household SES scores from CBS, which is based on household data concerning welfare (a combination of income and assets), highest level of education, and recent labour participation (Statistics Netherlands, 2024b). Household SES was categorized into quintiles, with higher quintiles representing higher household SES. Urbanicity was defined by the number of addresses in a 1 km^2^ circular buffer around the residential address. Urbanicity was categorized into non-urban (< 500 addresses/km^2^), limited urban (500–999 addresses/km^2^), moderately urban (1000–1499 addresses/km^2^), strong urban (1500–2499 addresses/km^2^), and very strong urban (≥ 2500 addresses/km^2^). For the purpose of stratified analyses, urbanicity was also dichotomized into rural to moderately urbanized areas (< 2000 addresses/km^2^) and highly urbanized areas (≥ 2000 addresses/km^2^). This approach is in line with previous research (van den Brekel et al., 2024).

### Statistical analysis

First, the distribution maps of green space at the neighbourhoodlevel in the Netherlands were created, for total green space, trees, shrubs, low vegetation, grass field, and agriculture, respectively. In the Netherlands, neighbourhoods (average area size: 3.1 km^2^) are geographically delineated areas within municipalities and include, on average, approximately 630 households (Timmermans et al., 2018).

Second, the distribution of the analytical sample was described in terms of socio-demographic and socio-economic characteristics, including age groups, sex, ethnicity, household SES, and urbanicity degree.

Third, the median (interquartile range) exposure of green space at the individual level was described by age groups, sex, ethnicity, quintiles of household SES, and urbanicity degree and stratified by green space type. The Kruskal–Wallis tests were conducted to examine the differences in green space density across each socio-demographic or socio-economic group. In the main analyses, green space exposure in 1000-m Euclidean buffers was used. To assess the robustness of our findings, sensitivity analyses included green space exposure in smaller (500 m) and larger (2000 m) Euclidean buffers.

Fourth, multiple linear regression models (based on Ordinary Least Squares (OLS)) were used to investigate the independent association of each factor with green space density, total for all types and for each type separately. Age groups, sex, ethnicity, household SES, and urbanicity degree were included as independent variables or covariates in the models. All records and datasets were anonymized. All statistical analyses were conducted in R software. Statistical significance was defined as *P* < 0.05 (2-sided).

## Results

Table 1 presents socio-demographic and socio-economic characteristics of 16,440,620 included residents in 2017. Children, adolescents, adults, and older adults consisted 12.9%, 7.3%, 61.4%, and 18.5% of the population, respectively. The percentage of adults was larger in highly urbanized areas than in rural to moderately urbanized areas. The number of males and females was balanced. A higher proportion of native ethnic Dutch residents was observed in rural to moderately urbanized areas compared to highly urbanized areas (89.2% vs. 73.6%). The proportion of people with higher household SES was higher in rural to moderately urbanized areas than in highly urbanized areas. More residents lived in neighbourhoods with an urbanicity degree of 1500–2499 addresses/km^2^ (26.0%) than neighbourhoods with other urbanicity levels.

**Table 1:** Socio-demographic and socio-economic characteristics of all included Dutch residents in 2017 (*n* = 16,440,620)

Characteristics	All, %	Rural to moderately urbanized areas (< 2000 addresses/km^2^), %	Highly urbanized areas (≥ 2000 addresses/km^2^), %
Age groups			
Children (1 to < 12 years)	12.9	13.1	12.4
Adolescents (12 to < 18 years)	7.3	7.9	6.0
Adults (18 to < 65 years)	61.4	59.8	64.6
Older adults (≥ 65 years)	18.5	19.2	17.0
Sex			
Males	49.7	49.9	49.2
Females	50.3	50.1	50.9
Ethnicity^a^			
Dutch	77.9	89.2	73.6
Turkish	2.3	1.3	5.3
Moroccan	2.2	1.2	5.2
Indonesian	2.1	1.9	3.1
Household SES^b^			
Quintile 1	19.4	14.9	29.1
Quintile 2	20.0	20.0	20.0
Quintile 3	20.3	21.9	16.8
Quintile 4	20.3	22.5	15.5
Quintile 5	20.1	20.8	18.7
Urbanicity degree (addresses/km^2^)			
Non-urban (< 500)	16.4	24.1	0.0
Limited urban (500–999)	17.0	25.0	0.0
Moderately urban (1000–1499)	19.4	28.5	0.0
Strong urban (1500–2499)	26.0	22.4	33.6
Very strong urban (≥ 2500)	21.2	0.0	66.4

^a^A total of 15.5% of the population belongs to other ethnicity groups and is not shown here

^b^Household socio-economic status (SES) score is developed by Statistics Netherlands based on standardized disposable income, taxable assets, highest level of education, and recent labour participation. A total of 1.8% of the population was missing in SES score. Quintile 1 is the lowest SES group

### Distribution of green space at the neighbourhood level

**Fig. 1:**
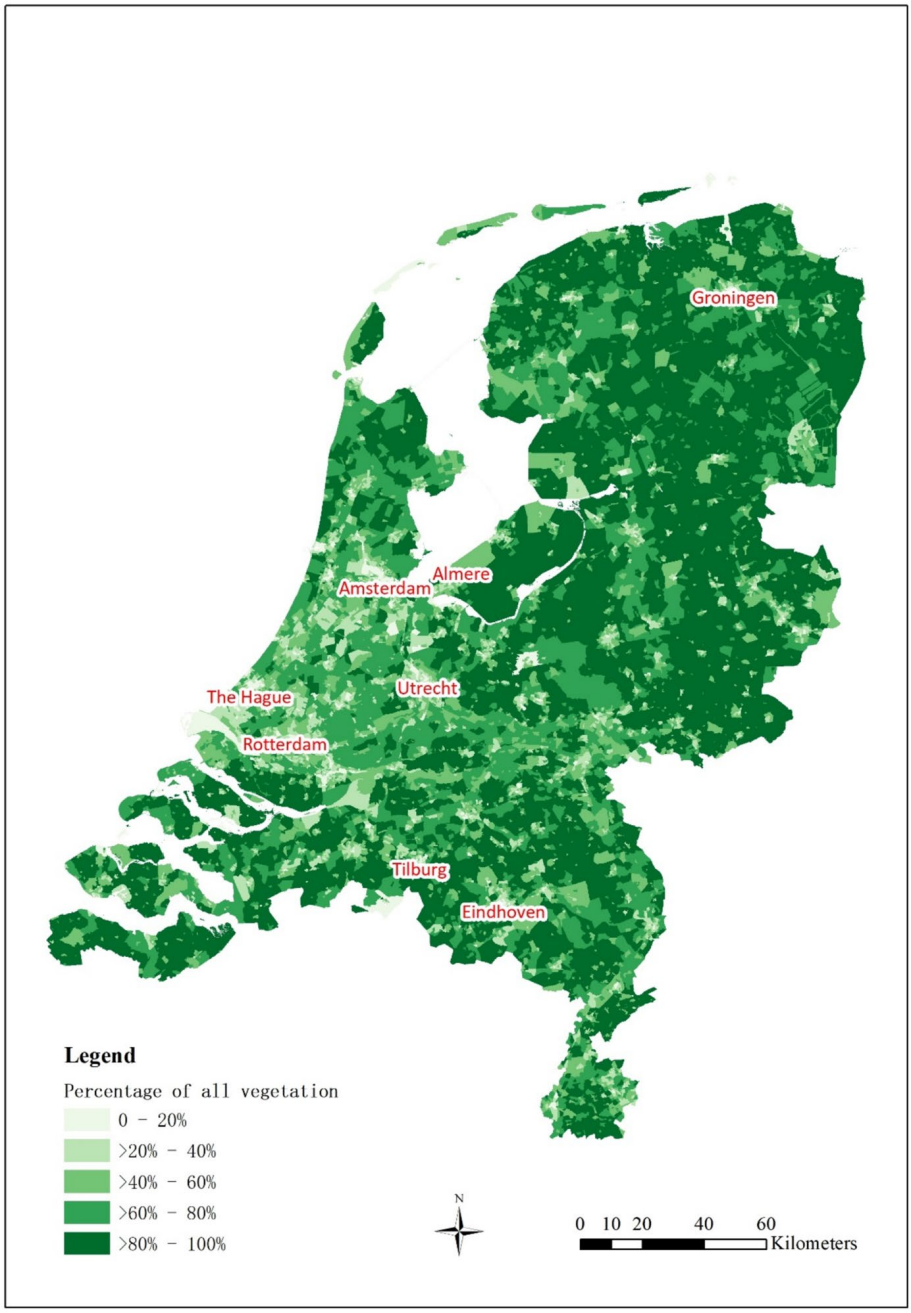
Distribution of green space (all vegetation including agriculture) at the neighbourhood level in the Netherlands, 2017. Cities with a population above 200,000 in 2017 were labelled

Figure 1 presents the distribution of green space at the neighbourhood level in the Netherlands in 2017. In general, residents living in the Netherlands had a high coverage of green space in their neighbourhoods. Most neighbourhoods in the north-eastern part of the Netherlands had more than eighty percent vegetation. Most neighbourhoods in cities had twenty to forty percent vegetation, except for the cities in middle-western areas like Amsterdam, Utrecht, Rotterdam, and The Hague. Neighbourhoods in centers of these big cities had less than twenty percent vegetation, while the peripheral neighbourhoods had more green space coverage.

A large proportion of the green space in the north-eastern and south-western parts of the Netherlands was agriculture (Appendix Fig. S1). There was barely any agriculture in cities and in the coastland. There were more trees in neighbourhoods in the eastern part of the Netherlands (more rural areas) than in neighbourhoods in the western part of the country (more urban areas) (Appendix Fig. S2). Neighbourhoods in cities in the west had less than twenty percent tree coverage, whereas this was higher in the east. On the contrary, there were more shrubs in the neighbourhoods in the western part of the Netherlands, especially in the coastland, than in the eastern part of the country (Appendix Fig. S3). The distribution of low vegetation was largely comparable to agriculture, except that the neighbourhoods in urban areas contained more low vegetation than agriculture (Appendix Fig. S4). This can mainly be attributed to grass field, another component of low vegetation. Neighbourhoods in highly urbanized areas had 16% to 36% grass field coverage while neighbourhoods in rural to moderately urbanized areas barely had grass field (Appendix Fig. S5). Neighbourhoods with the highest levels of grass field exposure were found in the coastland.

### Distribution of green space at the individual level

#### Availability of green space by age and sex

There were hardly any differences in green space exposure across age and sex groups. This was regardless of green space type (Appendix Figs. S6 and S7). After controlling for multiple covariates, the green space density around their residence increased by 0.69% (95% CI: 0.67% to 0.70%; *P* < 0.001) comparing older adults to children; and increased by 0.03% (95% CI: 0.02% to 0.04%; *P* < 0.001) comparing females to males (Table 2).

**Table 2:** Linear relation between socio-demographic and socio-economic characteristics and green space density by types within 1000-m Euclidean buffer zones around residential addresses in 2017 in the Netherlands (*n* = 16,440,620) ^a^

	β (95% CI)				
Characteristics	Total green space density	Tree density	Shrubs density	Low vegetation density	Agriculture density
Age groups					
Children (1 to < 12 years)	Ref	Ref	Ref	Ref	Ref
Adolescents (12 to < 18 years)	**0.23 (0.21, 0.24)**	**0.16 (0.14, 0.18)**	**0.021 (0.019, 0.024)**	0.02 (−0.01, 0.03)	**0.38 (0.35, 0.41)**
Adults (18 to < 65 years)	** − 0.03 (− 0.04, − 0.02)**	**0.26 (0.25, 0.28)**	**0.002 (0.001, 0.004)**	** − 0.38 (− 0.40, − 0.37)**	** − 0.10 (− 0.12, − 0.08)**
Older adults (≥ 65 years)	**0.69 (0.67, 0.70)**	**1.13 (1.12, 1.15)**	**0.078 (0.076, 0.080)**	** − 0.78 (− 0.79, −0.76)**	** − 0.47 (−0.50, − 0.45)**
Sex					
Males	Ref	Ref	Ref	Ref	Ref
Females	**0.03 (0.02, 0.04)**	**0.013 (0.005, 0.021)**	**0.008 (0.007, 0.009)**	** − **0.01 (**− **0.02, − 0.002)	** − 0.04 (− 0.05, − 0.02)**
Ethnicity ^b^					
Dutch	Ref	Ref	Ref	Ref	Ref
Turkish	** − 0.51 (− 0.53, − 0.48)**	**0.24 (0.22, 0.27)**	** − 0.223 (− 0.226, − 0.220)**	** − 0.06 (− 0.09, − 0.03)**	** − 1.39 (− 1.43, − 1.34)**
Moroccan	** − 0.04 (− 0.06, − 0.01)**	**0.17 (0.14, 0.19)**	** − 0.076 (− 0.079, − 0.072)**	**0.40 (0.37, 0.43)**	** − 0.98 (− 1.03, − 0.93)**
Indonesian	**0.61 (0.58, 0.63)**	**0.61 (0.58, 0.64)**	**0.091 (0.087, 0.094)**	** − 0.24 (− 0.27, −0.21)**	** − 1.35 (− 1.39, − 1.30)**
Household SES ^c^					
Quintile 1	Ref	Ref	Ref	Ref	Ref
Quintile 2	** − 0.07 (− 0.08, −0.06)**	** − 0.41 (− 0.42, − 0.39)**	**0.056 (0.055, 0.058)**	**0.37 (0.35, 0.38)**	**0.73 (0.71, 0.75)**
Quintile 3	**0.03 (0.02, 0.04)**	** − 0.47 (− 0.49, − 0.46)**	**0.101 (0.100, 0.103)**	**0.513 (0.499, 0.527)**	**1.07 (1.05, 1.10)**
Quintile 4	**0.06 (0.05, 0.07)**	** − 0.67 (− 0.68, − 0.65)**	**0.134 (0.132, 0.136)**	**0.74 (0.72, 0.75)**	**1.69 (1.67, 1.72)**
Quintile 5	**0.39 (0.38, 0.40)**	** − 0.23 (− 0.25, − 0.22)**	**0.186 (0.184, 0.188)**	**0.24 (0.23, 0.26)**	**0.89 (0.87, 0.91)**
Urbanicity degree (addresses/km^2^)					
Non-urban (< 500)	Ref	Ref	Ref	Ref	Ref
Limited urban (500–999)	** − 12.37 (− 12.38, − 12.35)**	** − 4.00 (− 4.01, − 3.98)**	** − 0.142 (− 0.144, − 0.141)**	** − 15.18 (− 15.20, − 15.17)**	** − 29.47 (− 29.49, − 29.45)**
Moderately urban (1000–1499)	** − 19.19 (− 19.21, − 19.18)**	** − 5.85 (− 5.87, − 5.84)**	** − 0.308 (− 0.310, − 0.307)**	** − 22.68 (− 22.70, − 22.67)**	** − 44.74 (− 44.76, − 44.72)**
Strong urban (1500–2499)	** − 24.87 (− 24.88, − 24.85)**	** − 6.82 (− 6.84, − 6.81)**	** − 0.444 (− 0.446, − 0.442)**	** − 28.88 (− 28.89, − 28.87)**	** − 56.39 (− 56.41, − 56.36)**
Very strong urban (≥ 2500)	** − 31.00 (− 31.01, − 30.99)**	** − 7.57 (− 7.59, − 7.56)**	** − 0.766 (− 0.768, − 0.764)**	** − 36.22 (− 36.24, − 36.21)**	** − 64.07 (− 64.09, − 64.04)**

^a^The multiple linear regression models included all characteristic variables listed in the table. The estimates presented in bold are statistically significant at *P* < 0.001

^b^A total of 15.5% of the population belongs to other ethnicity groups and is not shown here

^c^Household socio-economic status (SES) score is developed by Statistics Netherlands based on standardized disposable income, taxable assets, highest level of education, and recent labour participation. A total of 1.8% of the population was missing in SES score. Quintile 1 is the lowest SES group

### Availability of green space by ethnicity

**Fig. 2:**
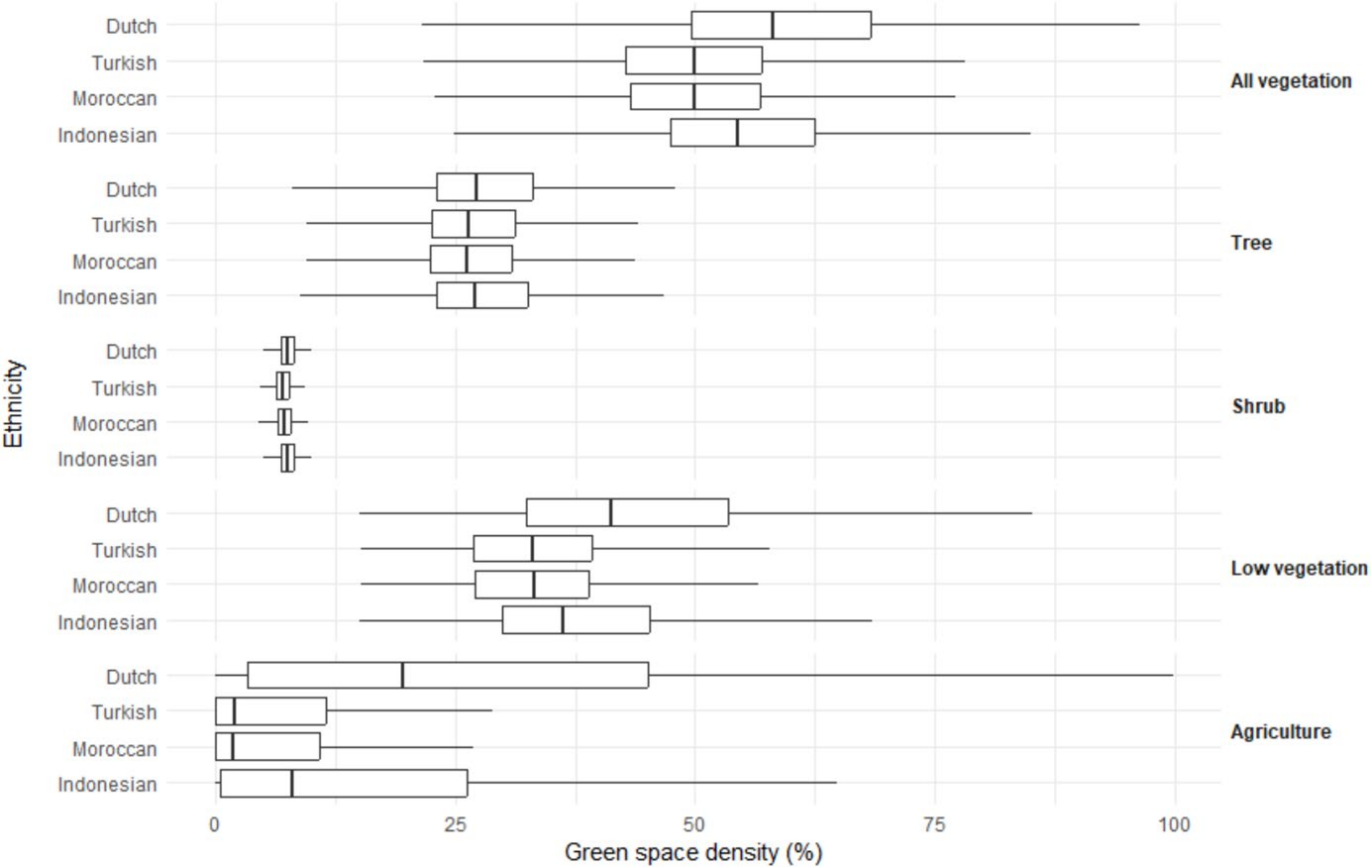
Boxplot of green space density by type and ethnicity within 1000-m Euclidean buffer zones around residential addresses in 2017 in the Netherlands (*n* = 13,894,514). The Kruskal–Wallis tests were conducted to examine the ethnic differences in green space density by type, respectively. Results were all significant at *P* < 0.001

**Fig. 3:**
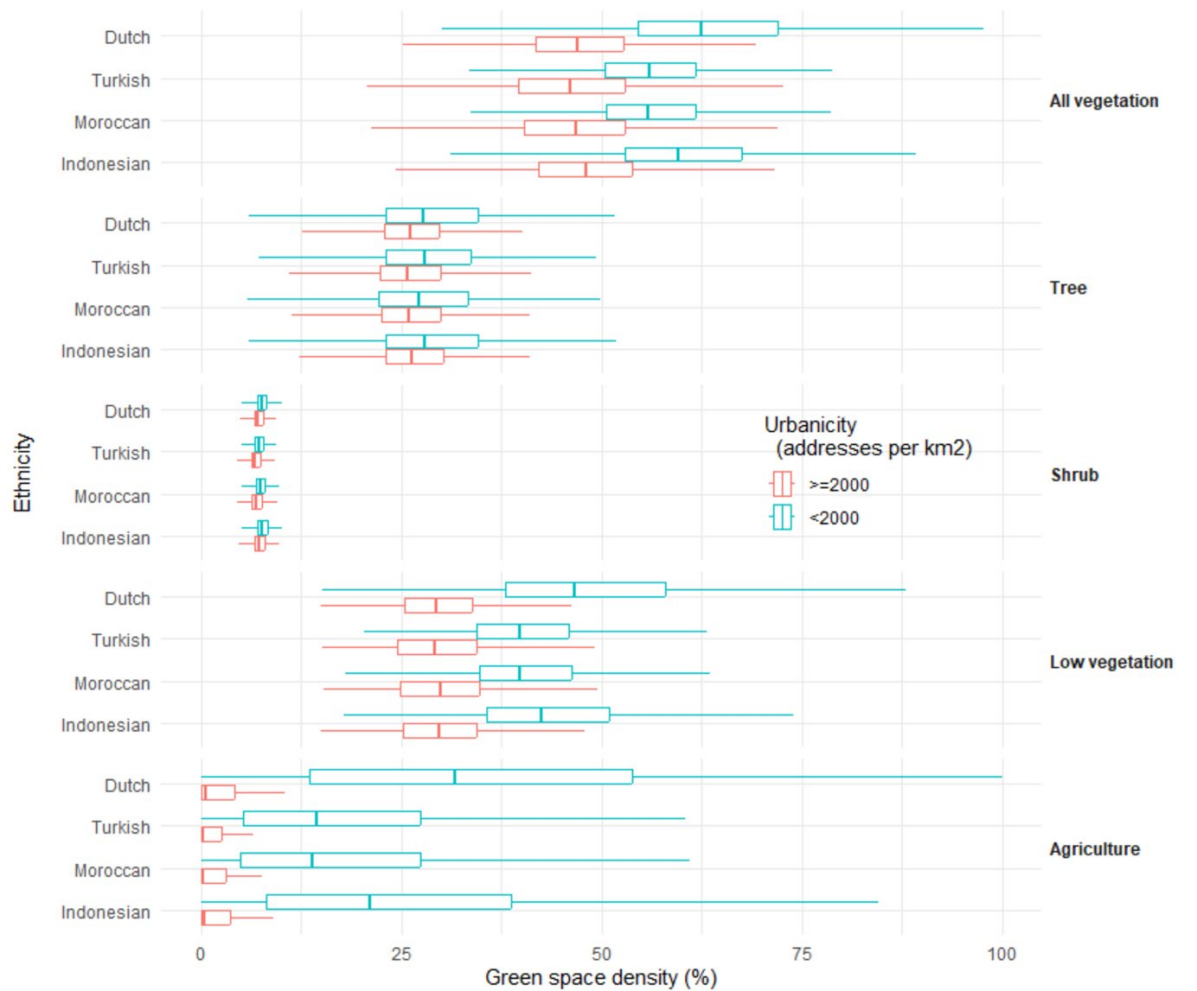
Boxplot of green space density by type and ethnicity within 1000-m Euclidean buffer zones around residential addresses in 2017 in the Netherlands stratified by urbanicity degree (*n* = 13,894,453). The Kruskal–Wallis tests were conducted to examine the ethnic differences in green space density by type within each urbanicity category, respectively. Results were all significant at *P* < 0.001

The ethnic Dutch (58.1%) and Indonesian (54.5%) had more green space density around their residence than Turkish (50.0%) and Moroccan (50.0%). This difference mainly originated from the difference in exposure to low vegetation (Fig. 2). When analyses were stratified by urbanicity (Fig. 3), the differences in the availability of green space between ethnic groups remained in the rural to moderately urbanized areas (low vegetation: 49.0%, 44.5%, 40.9%, and 41.1% for ethnic Dutch, Indonesian, Turkish, and Moroccan, respectively). However, in highly urbanized areas, differences between ethnic groups were much smaller (low vegetation: 30.0%, 30.2%, 29.9%, and 30.2% for ethnic Dutch, Indonesian, Turkish, and Moroccan, respectively). After adjustment for multiple covariates (Table 2), ethnic Indonesian had more green space density around their residence (β: 0.61%; 95% CI: 0.58% to 0.63%; *P* < 0.001), while Turkish (β: − 0.51%; 95% CI: − 0.53% to − 0.48%; *P* < 0.001) and Moroccan (β: − 0.04%; 95% CI: − 0.06% to − 0.01%; *P* < 0.001) had less green space density than ethnic Dutch. OLS indicated different results for tree density (Table 2), ethnic Turkish (β: 0.24%; 95% CI: 0.22% to 0.27%; *P* < 0.001), Moroccan (β: 0.17%; 95% CI: 0.14% to 0.19%; *P* < 0.001), and Indonesian (β: 0.61%; 95% CI: 0.58% to 0.64%; *P* < 0.001) all had more tree density around their residence than ethnic Dutch.

### Availability of green space by household SES

**Fig. 4:**
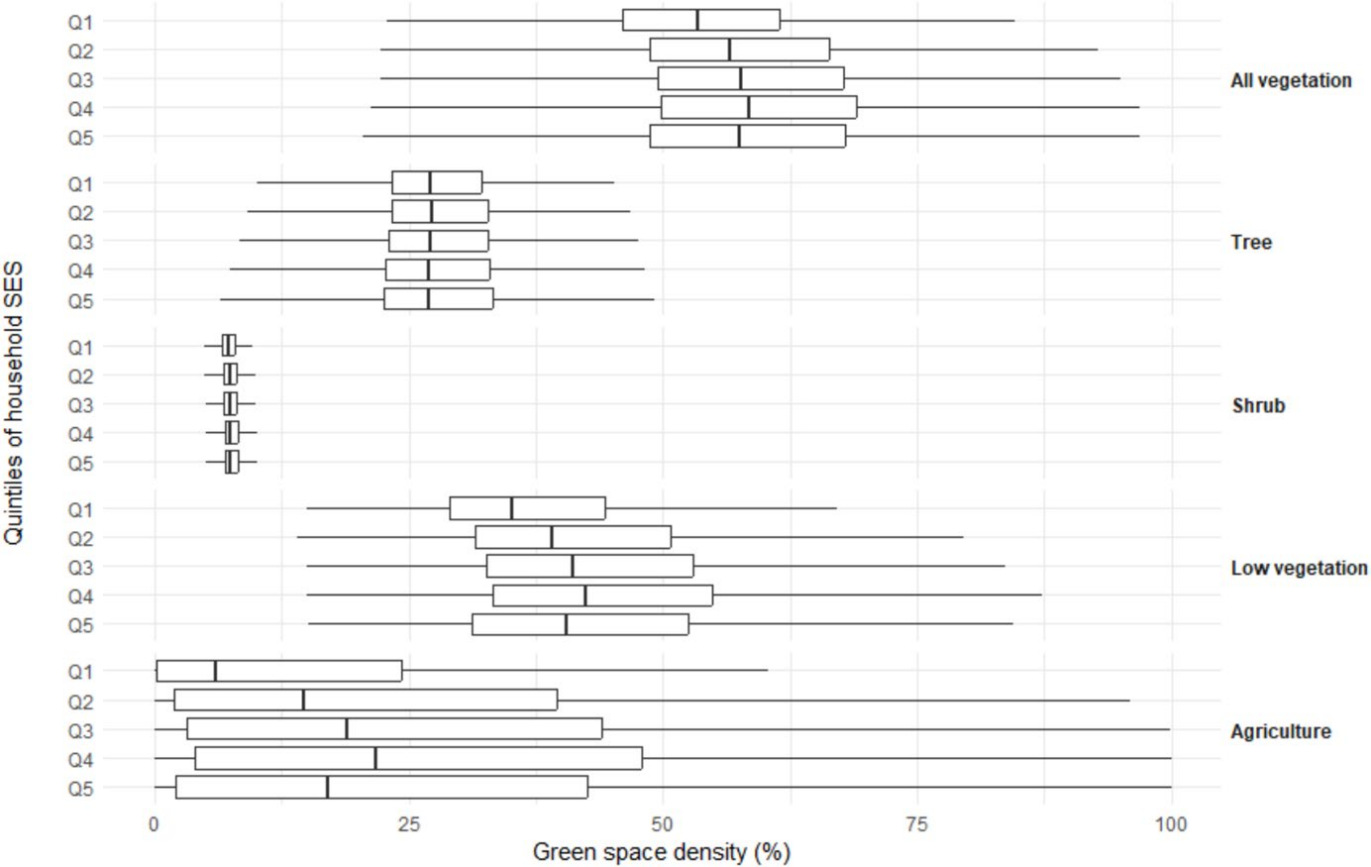
Boxplot of green space density by type and household socio-economic status (SES) score within 1000-m Euclidean buffer zones around residential addresses in 2017 in the Netherlands (*n* = 16,145,371). Household SES score is developed by Statistics Netherlands based on standardized disposable income, taxable assets, highest level of education, and recent labour participation. Q1 is the lowest SES group. The Kruskal–Wallis tests were conducted to examine the SES differences in green space density by type, respectively. Results were all significant at *P* < 0.001

**Fig. 5:**
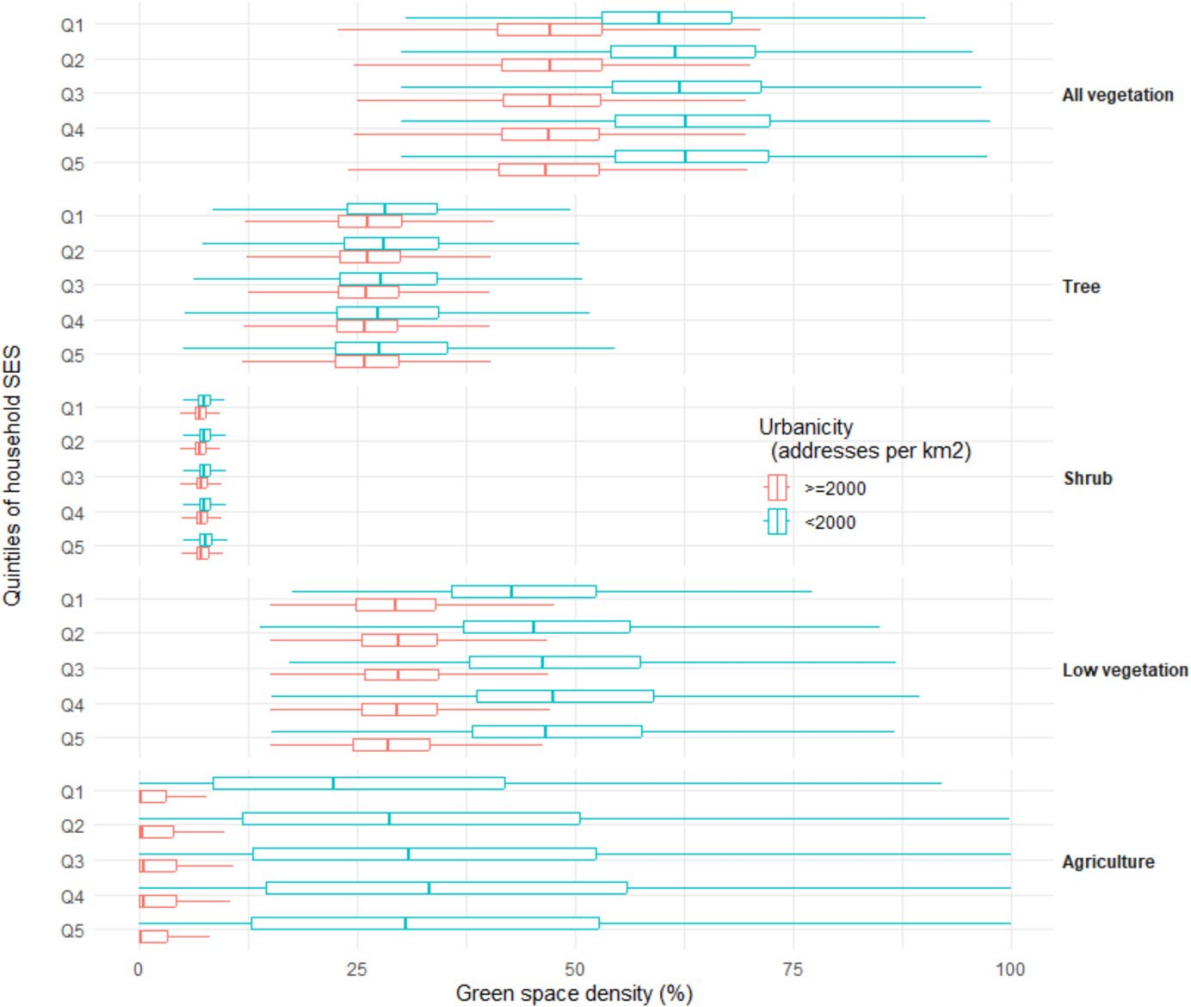
Boxplot of green space density by type and household socio-economic status (SES) score within 1000-m Euclidean buffer zones around residential addresses in 2017 in the Netherlands stratified by urbanicity degree (*n* = 16,145,294). Household SES score is developed by Statistics Netherlands based on standardized disposable income, taxable assets, highest level of education, and recent labour participation. Q1 is the lowest SES group. The Kruskal–Wallis tests were conducted to examine the SES differences in green space density by type within each urbanicity category, respectively. Results were all significant at *P* < 0.001

Individuals with higher household SES gradually had more green space density around their residence (48.0%, 50.3%, 51.2%, and 52.0% from quintile 1 to quintile 4, respectively), while in the highest household SES level, the density was slightly lower again (51.7% for quintile 5). This difference mainly originated from low vegetation (Fig. 4). When stratified by urbanicity (Fig. 5), the differences in the availability of green space between SES quintile groups remained in the rural to moderately urbanized areas (low vegetation: 45.3%, 47.8%, 48.7%, 49.8%, and 48.8% for quintile 1 to quintile 5, respectively). In highly urbanized areas, however, differences between SES groups were smaller (low vegetation: 29.8%, 30.3%, 30.5%, 30.2%, and 29.4% for quintile 1 to quintile 5, respectively). After controlling for multiple covariates (Table 2), the differences in the green space density were − 0.07% (95% CI: − 0.08% to − 0.06%; *P* < 0.001), 0.03% (95% CI: 0.02% to 0.04%; *P* < 0.001), 0.06% (95% CI: 0.05% to 0.07%; *P* < 0.001), and 0.39% (95% CI: 0.38% to 0.40%; *P* < 0.001) comparing each quintile to the lowest quintile of household SES. OLS indicated that higher household SES were associated with lower tree density (Table 2).

### Availability of green space by urbanicity degree

Residents living in areas with higher urbanicity degrees were exposed to lower levels of green space (Appendix Fig. S8). This originated from differences in exposures to both tree and low vegetation. After controlling for multiple covariates (Table 2), the green space density around their residence decreased by, as compared to non-urban areas, − 12.37% (95% CI: − 12.38% to − 12.35%; *P* < 0.001) for limited urban areas, − 19.19% (95% CI: − 19.21% to − 19.18%; *P* < 0.001) for moderately urban areas, − 24.87% (95% CI: − 24.88% to − 24.85%; *P* < 0.001) for strong urban areas, and − 31.00% (95% CI: − 31.01% to − 30.99%; *P* < 0.001) for very strong urban areas.

Detailed descriptive statistics on socio-demographic and socio-economic differences in green space density exposure in 1000-m Euclidean buffers around residential addresses, are presented in Appendix Table S1. Furthermore, similar details, related to the sensitivity analyses in which green space density in 500-m and 2000-m Euclidean buffers are included, are presented in Appendix Tables S2 and S3. Results of the sensitivity analyses were largely in line with those of the main analyses.

## Discussion

In this study, the distribution of green space by all vegetation, trees, shrubs, low vegetation, grass field, and agriculture was mapped for the Netherlands in 2017. This study also assessed socio-demographic and socio-economic differences in the availability of green space in the Netherlands. Hardly any differences in green space density were found across age or sex groups. Ethnic Dutch (58.1%) and Indonesian (54.5%) had more green space coverage around residence than Turkish (50.0%) and Moroccan (50.0%). People with higher household SES gradually had slightly more green space coverage (48.0% to 52.0%), while in the highest household SES level, the coverage decreased a little (51.7%). Higher urbanicity levels were monotonously associated with lower green space exposure. These differences particularly originated from differences in the density of low vegetation, and observed differences between ethnic and SES groups originated mostly from differences in rural to moderately urbanized areas and less from differences in highly urbanized areas. For the results from OLS, it should be noted that although most estimates were statistically significant due to the large sample size, the interpretation of results should also take into account the practical significance. For example, even though the differences in green space density were statistically significant across age or sex groups, the absolute difference in the density could be as small as 0.03%, representing a merely 942 m^2^ difference in a 1000-m Euclidean buffer zone (3,141,590 m^2^) around residential addresses. This small difference in green space density may barely affect its utility and health effects, and may be considered practically insignificant.

The results regarding ethnic disparities in green space exposure indicated that the ethnic Dutch and Indonesian populations were exposed to higher levels of green space density than the Turkish and Moroccan populations, which are the three largest ethnic minority groups in the Netherlands. These findings are in line with international research indicating that ethnic minority groups are exposed to less green space in comparison to the majority population. For instance, an individual-level study of urban Canadians found that minority ethnic populations (Latin American, African Canadian, and South Asian) were exposed to less green space than the majority population (Pinault et al., 2021). In addition, two nationwide US studies consistently found that census tracts with higher percentages of Hispanics were exposed to less green space (Klompmaker et al., 2023; Wen et al., 2013). After controlling for multiple covariates, the current study showed that Indonesian had more total green space exposure than ethnic Dutch, and all minorities had more tree exposure than ethnic Dutch. The adjusted results of tree exposure differed from the unadjusted results. These findings should be interpreted with caution. On the one hand, causal inference is not the objective of the current study. The adjusted results regarding ethnic disparities in tree exposure may not reflect the true association, given potential unmeasured covariates, interactions, and non-linear relationships. On the other hand, the unadjusted results plainly described the distribution of tree exposure across ethnic groups in the Netherlands.

The current study found urban–rural differences in green space exposure by ethnic groups. The ethnic disparities were more obvious in rural to moderately urbanized areas than in highly urbanized areas (Fig. 3). A 2015–2019 US nationwide study also found urban–rural differences. A weak positive association was observed between percentages of non-Hispanic Blacks and green space exposures in urban tracts but not in rural tracts (Klompmaker et al., 2023).

The difference in exposure to green space density across different SES groups was also examined by previous Dutch studies. Kruize and colleagues explored the distribution of several environmental aspects among SES groups in Netherlands (Kruize, 2007; Kruize et al., 2007a, b). For instance, they found that people with higher income levels had more public green space exposure around residence (Kruize, 2007). The findings of the current study indicated that the positive association between SES and green space was not monotonous. The highest SES quintile had a slightly lower green space coverage compared to the fourth quintile. SES is defined in the current study as a combined measure of income, assets, education, and occupation. The results of the current study may be explained by the fact that people with lower SES and the highest SES were more likely to live in urban areas, and higher green space density was associated with a lower urbanicity degree. Van Velzen et al. assessed green space distribution around Dutch primary schools and found that schools in low SES neighbourhoods were exposed to lower levels of outdoor green space in comparison to high SES neighbourhoods (van Velzen & Helbich, 2023). In this study of Dutch primary schools, the percentage of non-Western migrants in the neighbourhood was associated with more outdoor green space, and no evidence was found for green space disparity across urbanicity levels (van Velzen & Helbich, 2023). Previous international studies confirmed that higher SES levels were associated with more green space (Klompmaker et al., 2023; Pinault et al., 2021; Schüle et al., 2019; P. Sun & Lu, 2022; Yi Sun et al., 2021; Wen et al., 2013; Yu et al., 2020; Zhuang et al., 2023). The current Dutch nationwide study and the 2015–2019 US nationwide study both found urban–rural differences in green space exposure by SES groups (Klompmaker et al., 2023).

Previous studies on socio-demographic and socio-economic differences in green space density in the Netherlands did not consider agricultural land as a component of green space exposure. For instance, Kruize and colleagues defined green space as parks, forests, recreational areas and nature (Kruize, 2007; Kruize et al., 2007a, b), and Van Velzen et al. used a green space measure based on grass field, shrubs, and trees (van Velzen & Helbich, 2023). Agriculture, however, is an important component of green space in the Netherlands as shown in the current study. First, agriculture contributes to a large proportion of green space in the Netherlands. Second, the identified ethnic and socio-economic differences in green space exposure originated from low vegetation, which consists of grass field and agriculture. Grass field was mostly present in urban areas and agriculture was mostly present in rural areas. In addition, stratified results showed that the identified differences were in rural to moderately urbanized areas, but not in highly urbanized areas. These findings indicate that the identified differences mostly originated from differences in the availability of agriculture, and suggest that it is important to consider agriculture in green space exposure measures in future research.

Green space exposure has been associated with multiple health benefits, including improved mental health, and lower risk of obesity, cardiometabolic diseases, and mortality (Ccami-Bernal et al., 2023; M. Liu et al., 2023; Markevych et al., 2017). One pathway through which green space exposure may influence health is increasing physical activity (Markevych et al., 2017). In the context of the Netherlands, parts of the agricultural land can also be actively used by residents. For instance, there are 159 recorded walking routes in agricultural areas named clog trail path (in Dutch: klompenpaden) (“Klompenpaden; de mooiste wandelroutes” 2024). Some of these areas are also popular among bikers, as there is an extensive cycle path network named cyclepath nodes (in Dutch: fietsknooppunten) of which many cross agricultural land (“Fietsknoop”, 2024). Therefore, agriculture may have a positive health effect by encouraging an active lifestyle. However, this positive health effect might not be equal for all ethnic groups since cycling is much more common among the ethnic Dutch (van Boggelen & Harms, 2006). On the other hand, agriculture has been a large contributor to multiple air pollutants in the Netherlands, especially ammonia, nitrogen oxides, and particulate matter with diameters < 10 µm (Vonk et al., 2018). Different from the decreasing effect of other types of green space on air pollution via deposition, agriculture increases air pollution and may have a detrimental effect on health. Since we have identified ethnic and SES differences in the availability of green space, consistent with previous studies, future studies on the health effects of green space should take ethnic and socioeconomic inequalities in green space exposure into account.

The current study has several strengths, including (1) the use of nationwide registry data, (2) inclusion of various types of green space, including agricultural land, and (3) an extensive examination of socio-demographic and socio-economic differences in green space exposure. There are also several limitations to consider when interpreting the current results. First, the cross-sectional design precludes us from making causal inference. Second, the availability of green space was only measured as density. Other important measures like accessibility and utility of green space were not included in the present study. Third, only the exposure at the residence level was included. Green space exposures (in socio-demographic and SES groups) at the workplace or during commute could be described in future studies. Fourth, although the green space density data from 2017 are currently the most up-to-date and detailed data that are available for linkage, the results might be somewhat outdated. Since 2017, some newly built areas have been developed and some redesign of the landscape might have taken place. We have not been able to take these changes into account within our current analyses.

Policymakers who strive for environmental justice could consider planting more low vegetation in certain neighbourhoods, specifically, those with a high percentage of ethnic Turkish and Moroccan, and with a low SES. In particular, more grass fields are needed in rural neighbourhoods with ethnic minorities and low SES. However, we only provided a nationwide view. To make practical policies, local governments should zoom in the data and find local differences in the availability of green space.

## Conclusion

In conclusion, this is the first study that described multiple aspects of the socio-demographic and socio-economic differences in green space density in the Netherlands. Small differences in the availability of green space were found across age and sex groups. Ethnic Dutch and Indonesian had more green space coverage around residence than Turkish and Moroccan. People with a higher household SES had gradually more green space coverage, while in the highest SES level, the coverage was slightly lower again. Higher urbanicity levels were monotonously associated with lower green space exposure. The differences between ethnic groups and household SES groups originated from differences in the availability of low vegetation, and were mostly present in rural to moderately urbanized areas.

## Supplementary Information

Below is the link to the electronic supplementary material.

Supplementary file1 (PDF 1.30 MB)

## Data Availability

Results are based on calculations using geodata and non-public microdata from Statistics Netherlands. Under certain conditions, the underlying encrypted microdata are accessible for statistical and scientific research. For further information contact microdata@cbs.nl. If verification of the analyses is desired and Statistics Netherlands provides access to the microdata, we will provide the R-scripts for cohort-building and analyses upon request to the corresponding author. The geodata can be requested from the Geoscience and Health Cohort Consortium (www.gecco.nl).
