# Supplementary material for: Socio-demographic and socio-economic differences in the availability of green space in the Netherlands
Source: Popul Environ. 2025 Jun 23;47(3):26. doi: 10.1007/s11111-025-00498-3 (PMC12183132; doi:10.1007/s11111-025-00498-3)
Supplement: Supplementary file 1 — Supplementary file1 (PDF 1.30 MB) [file 11111_2025_498_MOESM1_ESM.pdf]

Appendices

Socio-demographic and socio-economic differences in the availability of green space in the Netherlands

Mingwei Liu [1] , Erik J. Timmermans [1, *], Alfred Wagtendonk [2], Paul Meijer [1], Diederick E. Grobbee [1], Ilonca Vaartjes [1]

[1] Julius Center for Health Sciences and Primary Care, University Medical Center Utrecht, Utrecht University, Utrecht, the Netherlands

[2] Department of Epidemiology and Data Science, Amsterdam UMC, Vrije Universiteit Amsterdam, Amsterdam, the Netherlands

* Corresponding author: Erik J. Timmermans

Julius Center for Health Sciences and Primary Care, University Medical Center Utrecht, Utrecht University, Utrecht, the Netherlands. Internal mail no. Str6.131. P.O. Box 85500. 3508 GA Utrecht, the Netherlands. E-mail: E.J.Timmermans-5@umcutrecht.nl

Contents

Figure S1. Distribution of agriculture at the neighbourhood level in the Netherlands, 2017.... 3

Figure S2. Distribution of trees at the neighborhood level in the Netherlands, 2017. 4

Figure S3. Distribution of shrubs at the neighborhood level in the Netherlands, 2017. 5

Figure S4. Distribution of low vegetation (any green space lower than 1 meter including grass and agriculture) at the neighborhood level in the Netherlands, 2017. 6

Figure S5. Distribution of grass field at the neighborhood level in the Netherlands, 2017. 7

Figure S6. Boxplot of green space density by types and age groups within 1000-meter Euclidean buffer zones around residential addresses in 2017 in the Netherlands (n=16,440,620). 8

Figure S7. Boxplot of green space density by types and sex within 1000-meter Euclidean buffer zones around residential addresses in 2017 in the Netherlands (n=16,440,620). 8

Figure S8. Boxplot of green space density by types and urbanicity degree within 1000-meter Euclidean buffer zones around residential addresses in 2017 in the Netherlands (n=16,440,541). 9

Table S1. Socio-demographic and socio-economic differences in the availability of green space within 1000-meter Euclidean buffer zones around residential addresses in 2017 in the Netherlands (n=16,440,620) 10

Table S2. Socio-demographic and socio-economic differences in the availability of green space within 500-meter Euclidean buffer zones around residential addresses in 2017 in the Netherlands (n=16,440,620) 11

Table S3. Socio-demographic and socio-economic differences in the availability of green space within 1500-meter Euclidean buffer zones around residential addresses in 2017 in the Netherlands (n=16,440,620) 12

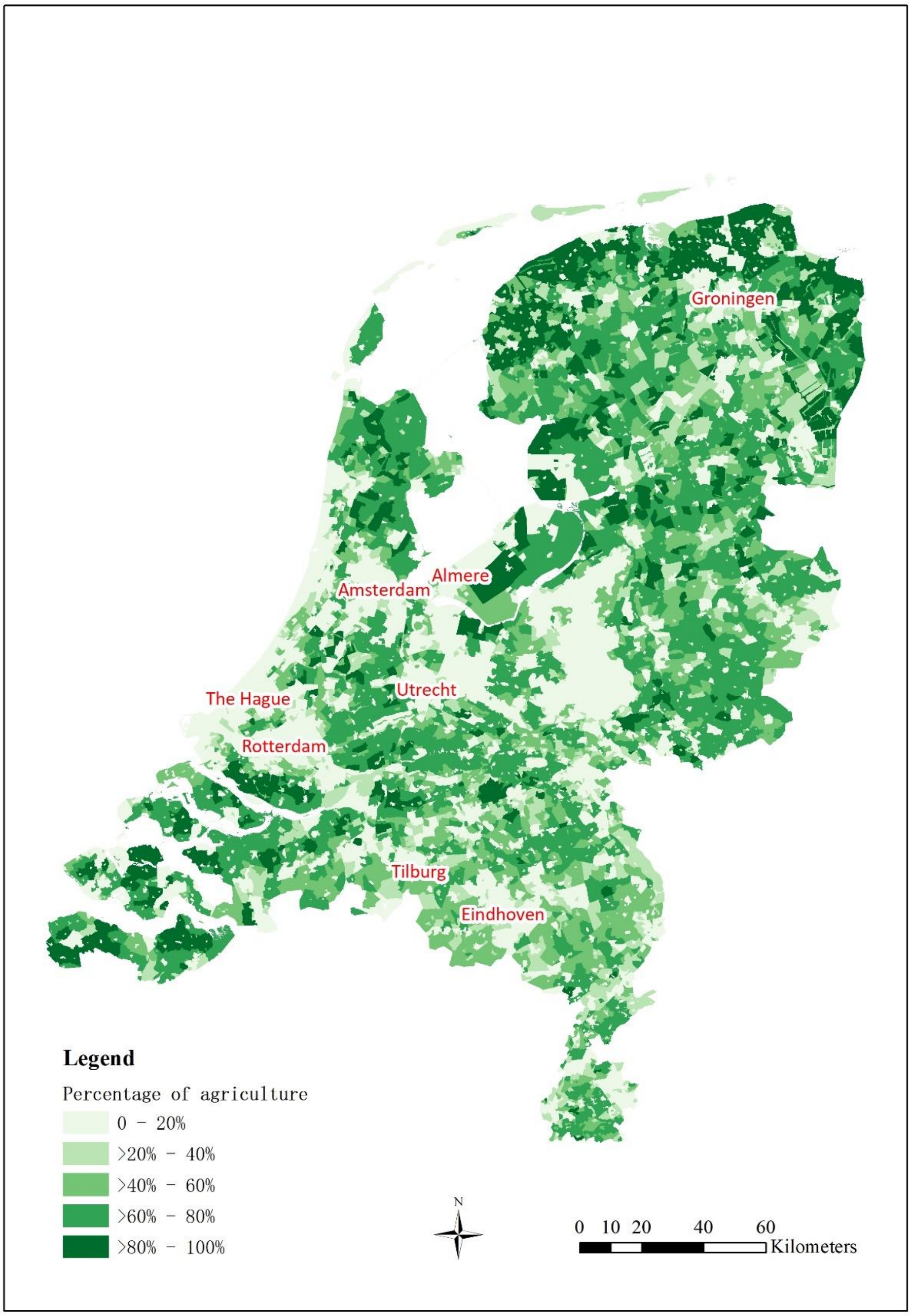

Figure S1. Distribution of agriculture at the neighbourhood level in the Netherlands, 2017. Cities with a population above 200,000 in 2017 were labeled.

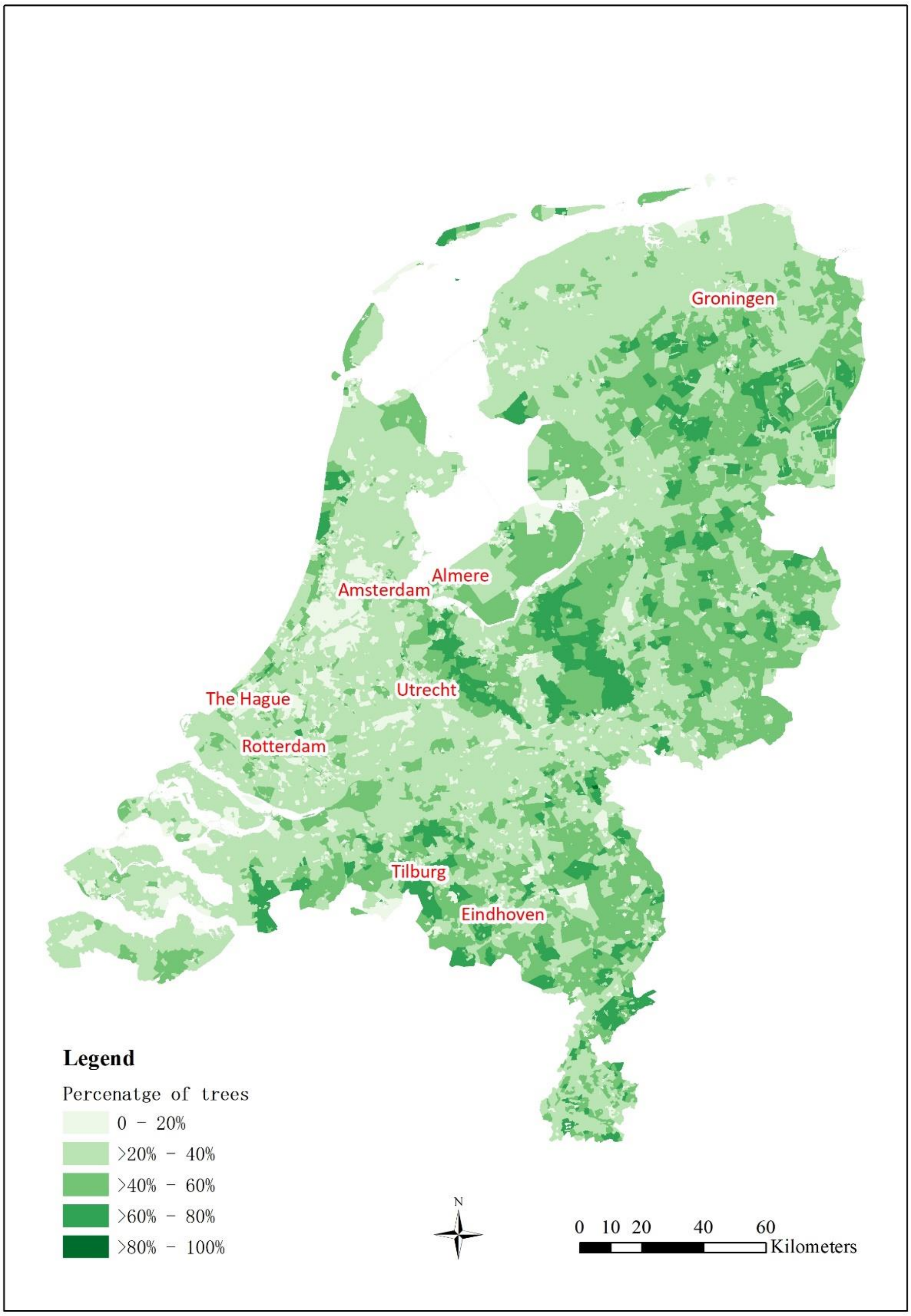

Figure S2. Distribution of trees at the neighborhood level in the Netherlands, 2017. Cities with a population above 200,000 in 2017 were labeled.

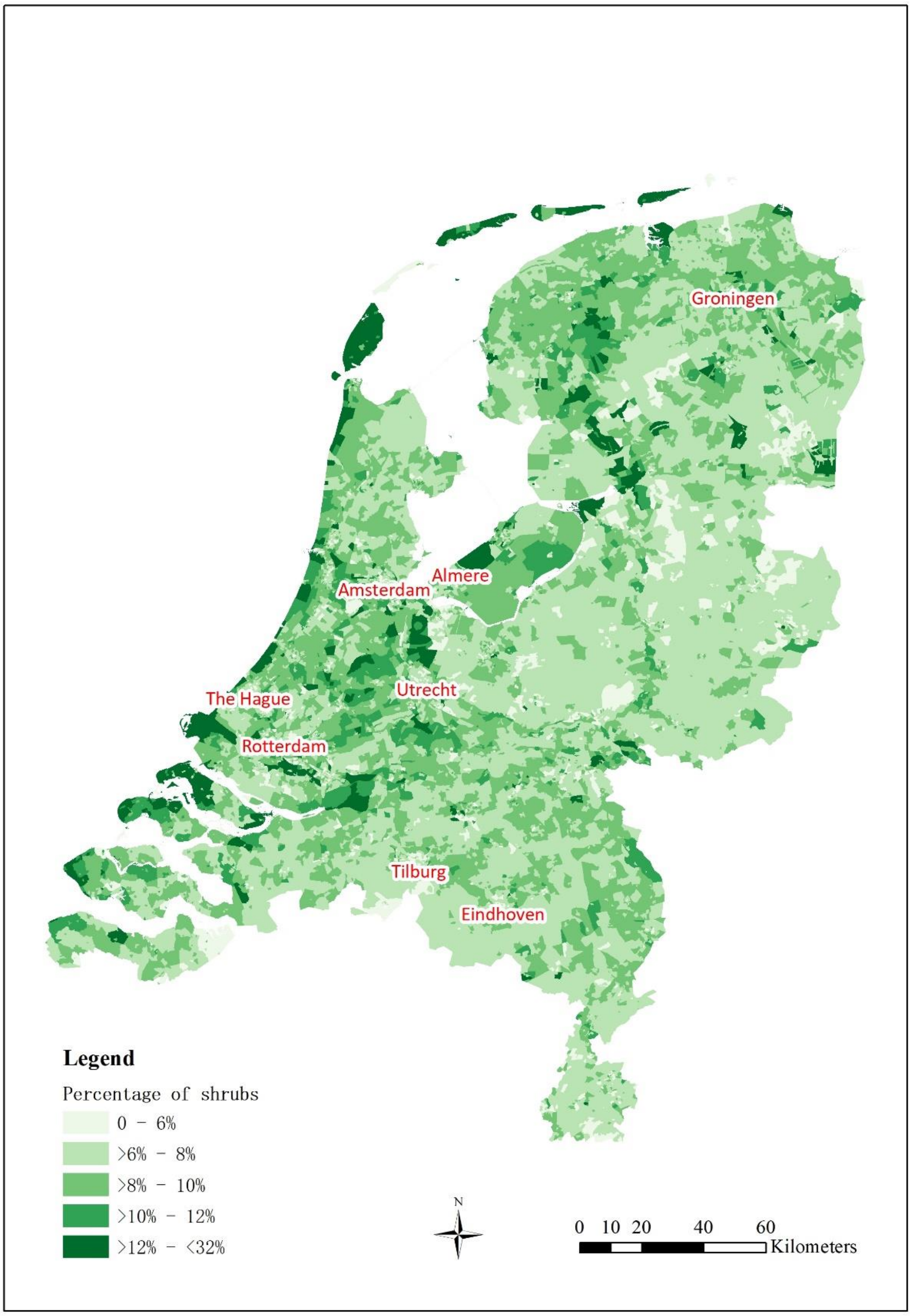

Figure S3. Distribution of shrubs at the neighborhood level in the Netherlands, 2017. Cities with a population above 200,000 in 2017 were labeled.

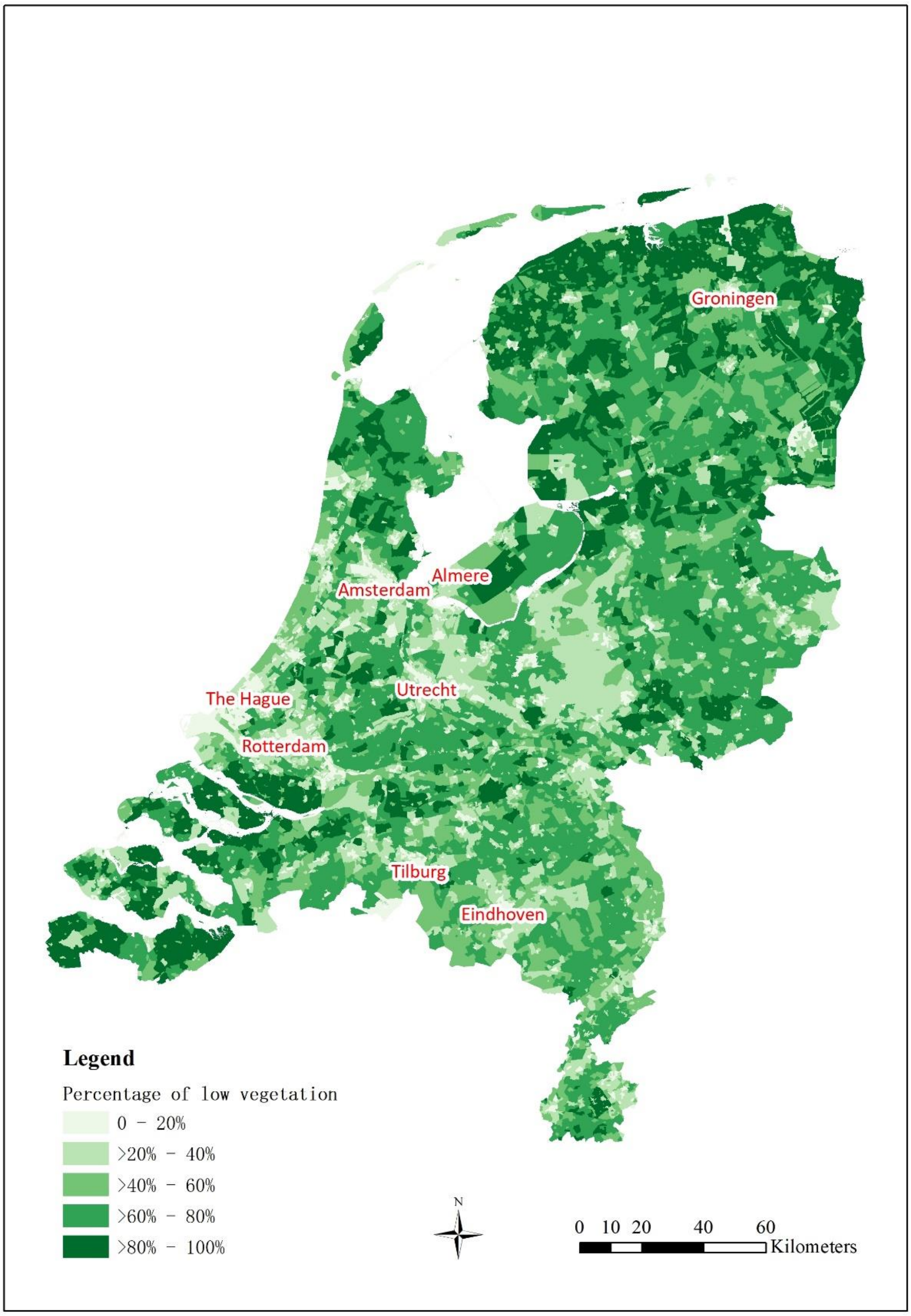

Figure S4. Distribution of low vegetation (any green space lower than 1 meter including grass and agriculture) at the neighborhood level in the Netherlands, 2017. Cities with a population above 200,000 in 2017 were labeled.

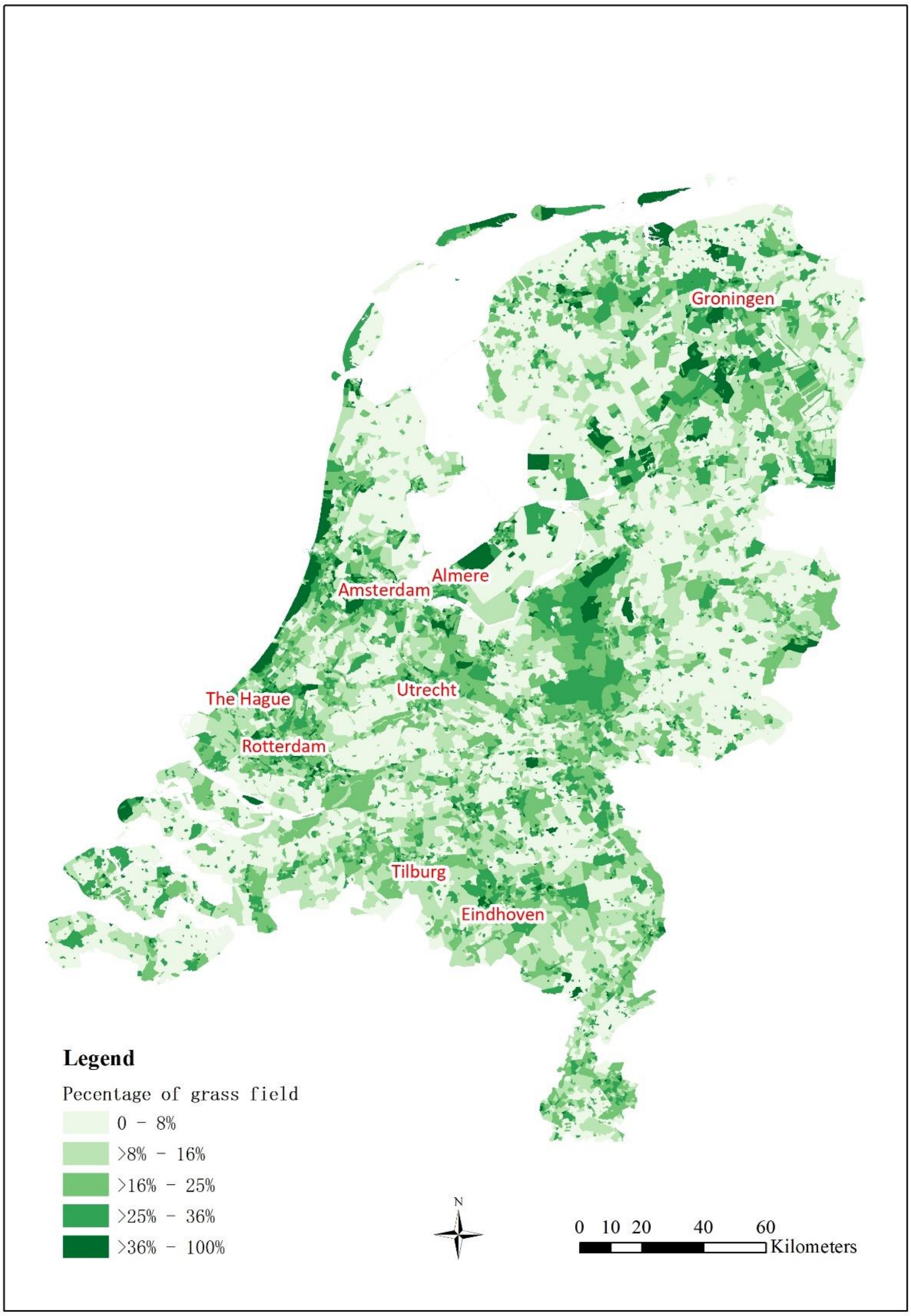

Figure S5. Distribution of grass field at the neighborhood level in the Netherlands, 2017. Cities with a population above 200,000 in 2017 were labeled.

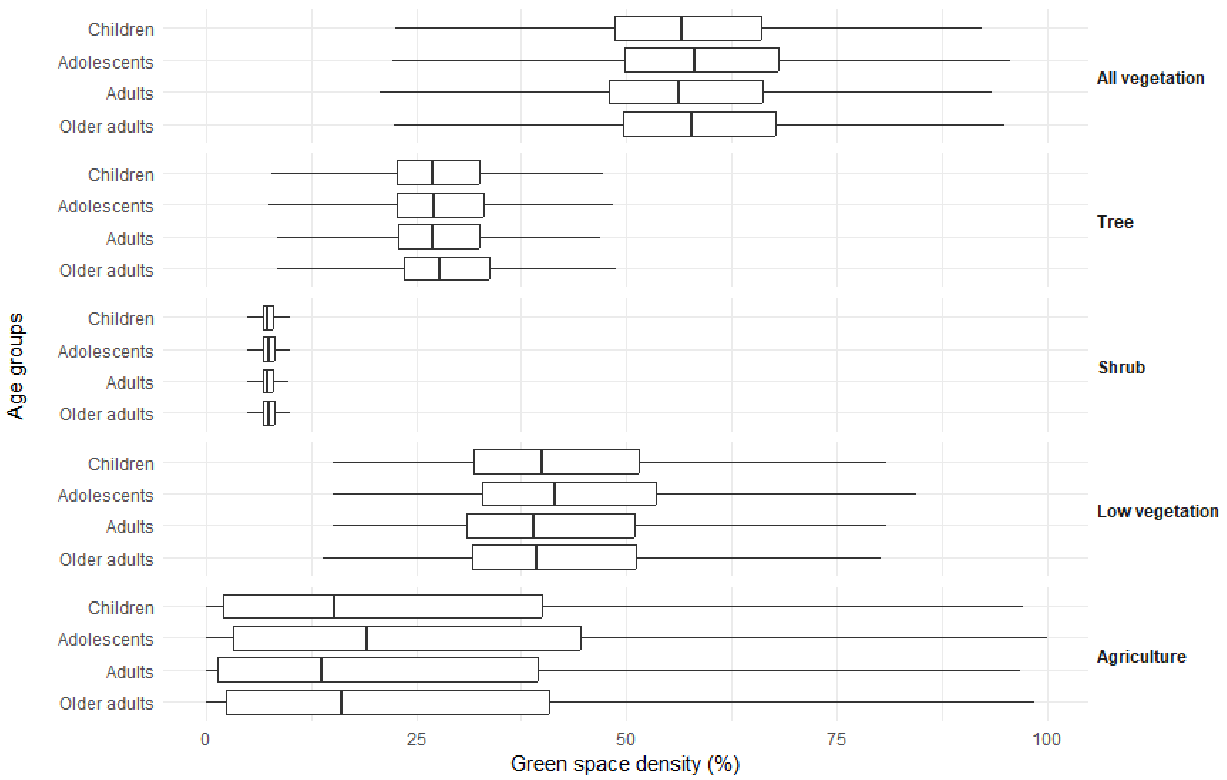

Figure S6. Boxplot of green space density by types and age groups within 1000-meter Euclidean buffer zones around residential addresses in 2017 in the Netherlands (n=16,440,620). Children: 1 to <12 years. Adolescents: 12 to <18 years. Adults: 18 to <65 years. Older adults: ≥65 years. The Kruskal-Wallis tests were conducted to examine differences in green space density by type across age groups, respectively. Results were all significant at P <0.001.

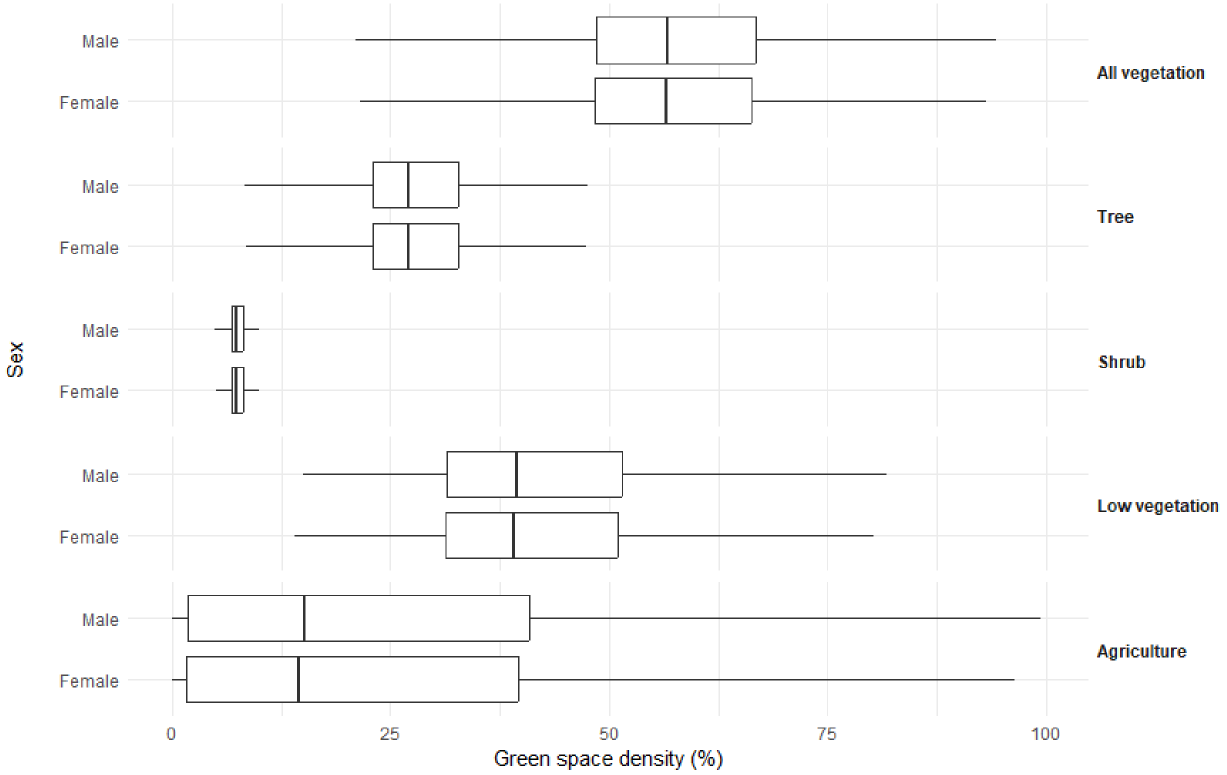

Figure S7. Boxplot of green space density by types and sex within 1000-meter Euclidean buffer zones around residential addresses in 2017 in the Netherlands (n=16,440,620). The Kruskal-Wallis tests were conducted to examine the sex differences in green space density by type, respectively. Results were all significant at P <0.001.

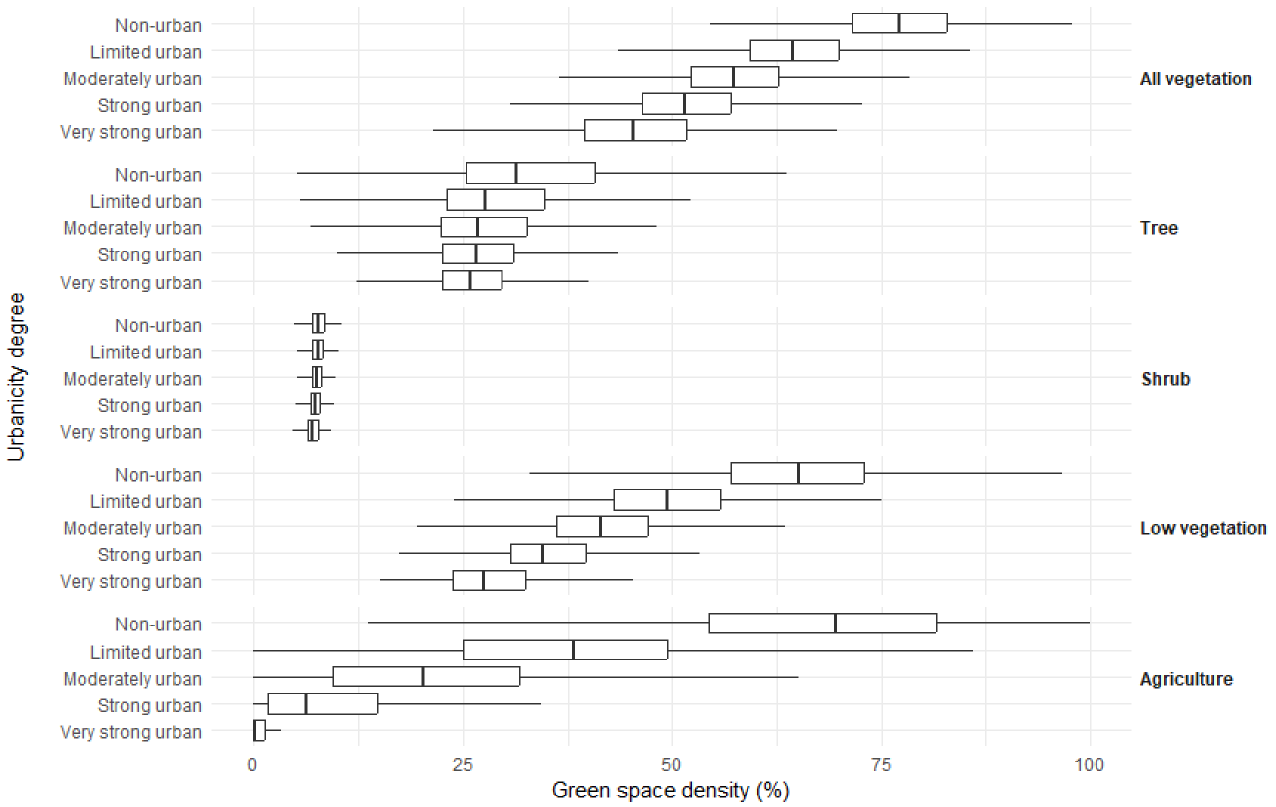

Figure S8. Boxplot of green space density by types and urbanicity degree within 1000-meter Euclidean buffer zones around residential addresses in 2017 in the Netherlands (n=16,440,541). Non-urban: <500 addresses/km^2. Limited urban: 500-999 addresses/km^2. Moderately urban: 1000-1499 addresses/km^2. Strong urban: 1500-2499 addresses/km^2. Very strong urban: ≥2500 addresses/km^2. The Kruskal-Wallis tests were conducted to examine differences in green space density by type across urbanicity categories, respectively. Results were all significant at P <0.001.

Table S1. Socio-demographic and socio-economic differences in the availability of green space within 1000-meter Euclidean buffer zones around residential addresses in 2017 in the Netherlands (n=16,440,620).

		Median (Quartile 1, Quartile 3) of density in percentage				
Characteristics	%	Total green space density	Tree density	Shrubs density	Low vegetation density	Agriculture density
Age groups						
Children (1 to <12 years)	12.85	56.61 (48.64, 66.09)	26.90 (22.65, 32.49)	7.40 (6.83, 8.08)	39.93 (31.87, 51.49)	15.24 (2.01, 40.05)
Adolescents (12 to <18 years)	7.32	58.01 (49.70, 68.07)	27.09 (22.78, 33.02)	7.45 (6.88, 8.13)	41.41 (32.83, 53.48)	19.14 (3.26, 44.48)
Adults (18 to <65 years)	61.36	56.17 (47.94, 66.12)	26.95 (22.90, 32.51)	7.38 (6.82, 8.06)	38.90 (30.93, 50.89)	13.82 (1.36, 39.51)
Older adults (≥65 years)	18.47	57.70 (49.59, 67.72)	27.75 (23.63, 33.66)	7.42 (6.87, 8.11)	39.36 (31.72, 51.12)	16.11 (2.50, 40.87)
Sex						
Males	49.66	56.76 (48.50, 66.80)	27.10 (22.98, 32.79)	7.40 (6.83, 8.08)	39.46 (31.38, 51.52)	15.16 (1.79, 40.85)
Females	50.34	56.55 (48.44, 66.37)	27.11 (23.01, 32.74)	7.40 (6.84, 8.08)	39.13 (31.27, 50.91)	14.50 (1.69, 39.60)
Ethnicity [1]						
Dutch	77.88	58.07 (49.59, 68.31)	27.19 (23.05, 33.03)	7.44 (6.88, 8.11)	41.21 (32.33, 53.49)	19.49 (3.28, 45.06)
Turkish	2.32	49.99 (42.84, 56.96)	26.34 (22.53, 31.18)	6.94 (6.41, 7.61)	33.04 (26.94, 39.27)	2.01 (0.04, 11.60)
Moroccan	2.19	49.95 (43.19, 56.78)	26.28 (22.40, 30.96)	7.12 (6.48, 7.76)	33.23 (27.14, 38.95)	1.81 (0.04, 10.78)
Indonesian	2.12	54.53 (47.43, 62.48)	27.12 (23.06, 32.52)	7.43 (6.86, 8.13)	36.21 (29.82, 45.30)	8.02 (0.53, 26.28)
Household SES [2]						
Quintile 1	19.40	53.41 (45.99, 61.44)	27.13 (23.32, 32.09)	7.22 (6.67, 7.87)	35.15 (28.97, 44.22)	5.93 (0.22, 24.29)
Quintile 2	19.97	56.58 (48.65, 66.29)	27.28 (23.30, 32.72)	7.38 (6.82, 8.05)	39.05 (31.46, 50.71)	14.62 (1.87, 39.49)
Quintile 3	20.25	57.72 (49.52, 67.69)	27.13 (23.01, 32.83)	7.43 (6.87, 8.11)	41.06 (32.62, 53.02)	18.95 (3.20, 44.02)
Quintile 4	20.27	58.51 (49.86, 68.93)	26.94 (22.70, 32.90)	7.47 (6.91, 8.16)	42.44 (33.18, 54.81)	21.79 (3.94, 47.78)
Quintile 5	20.10	57.52 (48.70, 67.97)	26.91 (22.51, 33.19)	7.49 (6.92, 8.19)	40.43 (31.28, 52.52)	17.05 (2.07, 42.61)
Urbanicity degree (addresses/km^2)						
Non-urban (<500)	16.42	77.16 (71.45, 82.76)	31.43 (25.36, 40.71)	7.72 (7.06, 8.47)	65.14 (56.93, 72.88)	69.51 (54.41, 81.53)
Limited urban (500-999)	16.98	64.43 (59.32, 69.83)	27.73 (23.07, 34.72)	7.62 (7.06, 8.30)	48.50 (43.06, 55.83)	38.24 (25.07, 49.45)
Moderately urban (1000-1499)	19.42	57.35 (52.19, 62.68)	26.74 (22.37, 32.70)	7.47 (6.97, 8.09)	41.43 (36.09, 47.09)	20.17 (9.51, 31.77)
Strong urban (1500-2499)	25.95	51.51 (46.43, 56.93)	26.55 (22.63, 31.04)	7.34 (6.82, 7.96)	34.60 (30.70, 39.73)	6.37 (1.66, 14.76)
Very strong urban (≥2500)	21.23	45.42 (39.58, 51.63)	25.89 (22.63, 29.58)	7.00 (6.48, 7.64)	27.47 (23.77, 32.39)	0.12 (0, 1.35)

[1] A total of 15.49% of the population belongs to other ethnicity groups and is not shown here.

[2] Household socio-economic status (SES) score is developed by Statistics Netherlands based on standardized disposable income, taxable assets, highest level of education, and recent labor participation. A total of 1.80% of the population was missing in SES score. Quintile 1 is the lowest SES group.

Table S2. Socio-demographic and socio-economic differences in the availability of green space within 500-meter Euclidean buffer zones around residential addresses in 2017 in the Netherlands (n=16,440,620).

Characteristics	%	Median (Quartile 1, Quartile 3) of density in percentage				
		Total green space density	Tree density	Shrubs density	Low vegetation density	Agriculture density
Age groups						
Children (1 to <12 years)	12.85	50.39 (42.59, 59.60)	24.04 (19.52, 29.38)	7.21 (6.54, 7.96)	33.94 (27.50, 43.88)	4.37 (0.08, 25.06)
Adolescents (12 to <18 years)	7.32	51.64 (43.60, 61.69)	24.23 (19.68, 29.80)	7.30 (6.62, 8.04)	35.00 (28.16, 45.81)	6.68 (0.16, 30.00)
Adults (18 to <65 years)	61.36	50.19 (42.16, 59.79)	24.25 (19.89, 29.54)	7.21 (6.54, 7.95)	33.26 (26.80, 43.31)	3.58 (0.08, 24.75)
Older adults (≥65 years)	18.47	51.51 (43.66, 61.27)	25.13 (20.79, 30.62)	7.26 (6.61, 8.00)	33.32 (27.28, 43.07)	4.54 (0.08, 25.38)
Sex						
Males	49.66	50.67 (42.62, 60.42)	24.38 (19.98, 29.76)	7.23 (6.56, 7.97)	33.62 (27.12, 43.85)	4.29 (0.08, 26.02)
Females	50.34	50.47 (42.56, 59.97)	24.40 (20.02, 29.72)	7.23 (6.56, 7.97)	33.35 (27.03, 43.21)	3.82 (0.08, 24.59)
Ethnicity [1]						
Dutch	77.88	51.56 (43.44, 61.77)	24.41 (20.02, 29.84)	7.29 (6.63, 8.02)	34.63 (27.68, 45.60)	6.69 (0.16, 30.16)
Turkish	2.32	45.09 (37.93, 52.26)	23.99 (19.91, 28.98)	6.65 (6.02, 7.35)	29.38 (24.13, 34.44)	0.08 (0.00, 2.86)
Moroccan	2.19	45.41 (38.45, 52.23)	24.13 (19.98, 28.97)	6.77 (6.08, 7.47)	29.54 (34.35, 34.31)	0.08 (0.00, 2.31)
Indonesian	2.12	49.17 (41.81, 57.28)	24.63 (20.06, 29.82)	7.26 (6.58, 8.03)	31.51 (26.03, 38.80)	0.80 (0.00, 13.37)
Household SES [2]						
Quintile 1	19.40	47.95 (40.55, 55.67)	24.64 (20.55, 29.52)	6.96 (6.31, 7.66)	30.73 (25.36, 37.31)	0.40 (0.00, 10.27)
Quintile 2	19.97	50.28 (42.58, 59.41)	24.57 (20.36, 29.69)	7.18 (6.53, 7.90)	33.08 (27.04, 42.45)	3.66 (0.08, 23.48)
Quintile 3	20.25	51.18 (43.28, 60.93)	24.35 (19.99, 29.65)	7.26 (6.61, 7.99)	34.47 (27.83, 44.89)	6.21 (0.16, 28.72)
Quintile 4	20.27	52.02 (43.66, 62.64)	24.10 (19.60, 29.63)	7.34 (6.67, 8.08)	35.73 (28.30, 47.33)	8.44 (0.16, 33.90)
Quintile 5	20.10	51.74 (43.18, 62.47)	24.09 (19.34, 29.93)	7.42 (6.73, 8.17)	34.70 (27.29, 45.95)	6.04 (0.08, 29.92)
Urbanicity degree (addresses/km^2)						
Non-urban (<500)	16.42	71.18 (62.84, 79.63)	27.40 (21.89, 35.65)	7.60 (6.89, 8.40)	57.43 (47.69, 68.02)	55.86 (37.40, 77.19)
Limited urban (500-999)	16.98	55.42 (48.83, 62.81)	23.91 (19.55, 29.87)	7.47 (6.86, 8.16)	39.98 (33.80, 47.12)	19.49 (7.64, 33.50)
Moderately urban (1000-1499)	19.42	50.39 (44.03, 57.14)	23.85 (19.19, 29.23)	7.30 (6.69, 8.01)	34.24 (29.16, 40.49)	6.44 (0.55, 18.23)
Strong urban (1500-2499)	25.95	46.26 (40.63, 52.55)	24.13 (19.77, 28.75)	7.12 (6.46, 7.84)	30.11 (26.18, 34.80)	0.40 (0.00, 5.33)
Very strong urban (≥2500)	21.23	41.94 (35.38, 49.49)	23.87 (20.08, 28.14)	6.81 (6.17, 7.50)	25.17 (21.05, 30.27)	0.00 (0.00, 0.16)

[1] A total of 7.84% of the population belongs to other ethnicity groups and is not shown here.

[2] Household socio-economic status (SES) score is developed by Statistics Netherlands based on standardized disposable income, taxable assets, highest level of education, and recent labor participation. A total of 1.80% of the population was missing in SES score. Quintile 1 is the lowest SES group.

Table S3. Socio-demographic and socio-economic differences in the availability of green space within 1500-meter Euclidean buffer zones around residential addresses in 2017 in the Netherlands (n=16,440,620).

		Median (Quartile 1, Quartile 3) of density in percentage				
Characteristics	%	Total green space density	Tree density	Shrubs density	Low vegetation density	Agriculture density
Age groups						
Children (1 to <12 years)	12.85	60.61 (52.48, 70.17)	28.88 (24.50, 34.64)	7.53 (6.99, 8.16)	44.37 (34.99, 56.07)	25.23 (6.94, 50.16)
Adolescents (12 to <18 years)	7.32	62.19 (53.68, 71.82)	29.15 (24.62, 35.36)	7.56 (7.02, 8.20)	45.95 (36.02, 57.82)	29.32 (9.15, 53.90)
Adults (18 to <65 years)	61.36	60.14 (51.71, 70.18)	28.89 (24.66, 34.66)	7.51 (6.98, 8.14)	43.30 (33.96, 55.48)	23.51 (5.59, 49.66)
Older adults (≥65 years)	18.47	62.05 (53.63, 71.81)	29.81 (25.29, 36.21)	7.54 (7.01, 8.20)	44.19 (35.09, 56.14)	26.64 (8.36, 51.88)
Sex						
Males	49.66	60.81 (52.32, 70.79)	29.07 (24.74, 35.01)	7.52 (6.98, 8.16)	43.98 (34.51, 56.09)	25.10 (6.56, 50.97)
Females	50.34	60.60 (52.28, 70.46)	29.07 (24.76, 34.96)	7.52 (6.99, 8.16)	43.66 (34.41, 55.63)	24.43 (6.42, 50.01)
Ethnicity [1]						
Dutch	77.88	62.45 (53.69, 72.21)	29.25 (24.82, 35.54)	7.55 (7.02, 8.19)	45.94 (35.80, 58.03)	29.98 (9.51, 54.82)
Turkish	2.32	53.27 (46.26, 59.53)	27.96 (24.23, 32.34)	7.12 (6.67, 7.74)	35.38 (28.94, 43.17)	6.79 (0.75, 20.30)
Moroccan	2.19	53.07 (46.25, 59.61)	27.56 (23.91, 32.13)	7.32 (6.75, 7.95)	35.60 (29.17, 42.94)	6.35 (0.71, 19.76)
Indonesian	2.12	58.08 (51.03, 66.13)	28.88 (24.71, 34.33)	7.56 (7.01, 8.21)	39.89 (32.67, 49.78)	16.28 (3.64, 36.32)
Household SES [2]						
Quintile 1	19.40	57.04 (49.59, 65.75)	28.88 (24.97, 33.82)	7.38 (6.87, 8.01)	38.62 (31.64, 49.32)	13.50 (2.33, 35.95)
Quintile 2	19.97	60.84 (52.60, 70.66)	29.25 (25.00, 34.98)	7.50 (6.98, 8.14)	43.80 (34.76, 55.81)	25.12 (7.06, 50.69)
Quintile 3	20.25	62.10 (53.61, 71.76)	29.16 (24.77, 35.19)	7.55 (7.02, 8.19)	45.82 (36.03, 57.75)	29.66 (9.47, 54.06)
Quintile 4	20.27	62.78 (53.90, 72.60)	29.02 (24.53, 35.39)	7.58 (7.04, 8.22)	47.02 (36.59, 58.95)	31.95 (10.39, 56.72)
Quintile 5	20.10	61.32 (52.41, 71.32)	28.96 (24.40, 35.71)	7.58 (7.04, 8.23)	44.56 (34.25, 56.35)	25.95 (6.63, 51.14)
Urbanicity degree (addresses/km^2)						
Non-urban (<500)	16.42	79.46 (74.41, 84.12)	34.05 (27.29, 43.78)	7.78 (7.14, 8.51)	67.97 (59.52, 74.94)	72.85 (58.67, 83.44)
Limited urban (500-999)	16.98	69.81 (65.06, 74.33)	30.79 (25.26, 38.54)	7.70 (7.15, 8.37)	55.12 (48.10, 61.98)	50.70 (36.16, 61.52)
Moderately urban (1000-1499)	19.42	62.14 (57.30, 66.97)	29.10 (24.28, 35.06)	7.59 (7.09, 8.16)	46.48 (40.54, 52.30)	31.87 (18.71, 43.45)
Strong urban (1500-2499)	25.95	55.49 (50.82, 60.48)	28.16 (24.32, 32.72)	7.46 (7.00, 8.07)	38.58 (34.04, 44.44)	14.54 (6.70, 25.09)
Very strong urban (≥2500)	21.23	48.43 (43.09, 53.98)	27.31 (24.25, 30.91)	7.19 (6.69, 7.79)	29.88 (25.90, 34.70)	1.31 (0.09, 5.51)

[1] A total of 7.84% of the population belongs to other ethnicity groups and is not shown here.

[2] Household socio-economic status (SES) score is developed by Statistics Netherlands based on standardized disposable income, taxable assets, highest level of education, and recent labor participation. A total of 1.80% of the population was missing in SES score. Quintile 1 is the lowest SES group.
